# Minimally invasive surgical correction technique of Pectus Arcuatum, case report of an innovative method for a rare chest wall malformation (Jerusalem technique)

**DOI:** 10.3389/fsurg.2025.1641213

**Published:** 2025-09-24

**Authors:** Daniella Moshe, Anas Salhab, Mordechai Shimonov, Firas Abu Akar

**Affiliations:** ^1^ Department of Surgery, The Edith Wolfson Medical Center, Holon, Tel Aviv, Israel; ^2^ Department of Thoracic Surgery, The Edith Wolfson Medical Center, Holon, Tel Aviv, Israel; ^3^ Gray Faculty of Medical and Health Sciences - Tel Aviv University, Tel Aviv-Yafo, Israel

**Keywords:** Pectus Arcuatum, chest wall malformation, minimally invasive surgery, Pectus surgery, Jerusalem technique, chest wall deformity correction, innovative surgical method

## Abstract

Pectus Arcuatum (PA) is a rare anterior chest wall deformity characterized by a deformation with potential psychological impact on patients and combining features of both pectus carinatum and pectus excavatum. Traditional corrective methods involve open surgical techniques, such as sternotomy, which often result in significant surgical scars. This case report presents two patients who underwent successful minimally invasive correction of PA using a modified sandwich method, referred to as the Jerusalem Technique. The innovative approach involved bilateral thoracoscopic-guided osteotomies and placement of one or more support bars, all performed through lateral incisions without midline exposure. Both patients demonstrated excellent cosmetic and functional outcomes, minimal postoperative pain, and no complications. As many patients seek surgery for cosmetic concerns, there is a growing interest in minimally invasive approaches, this technique offers an effective alternative to traditional open surgery, representing fully minimally invasive surgical correction for PA.

## Introduction

Pectus Arcuatum (PA) is a rare congenital chest wall deformity characterized by a distinctive figure of “number 7” or “L” shaped sternum, combining protrusion of the manubrium with depression of the sternal body. It has historically been labeled as Currarino-Silverman syndrome or “Pouter Pigeon Breast.” The condition is often misdiagnosed due to its overlapping features with more common chest wall malformations such as pectus excavatum (PE), pectus carinatum (PC) or a combined variant (1–3). While PA is typically asymptomatic, the significant cosmetic impact often drives patients to seek surgical correction. Traditional surgical approaches, including the Ravitch technique which was introduced by Mark M. Ravitch, as the first surgical correction for PA in 1952 and remains the standard treatment for most PA cases (4–7).

The Ravitch technique involves sternotomy and midline incisions that result in visible scarring and potential complications. Our literature review identified emerging hybrid surgical strategies that combine elements of minimally invasive correction techniques with the Ravitch procedure (8–10). In this paper, we introduce an innovative technique for complete minimally invasive surgery for PA repair and discuss two cases that were successfully performed as a preliminary experience in this technique (Table 1). This technique merges the principles of the sandwich method with thoracoscopic-guided osteotomy and substernal bar placement, offering an innovative and cosmetically superior solution.

**Table 1:** Timeline.

Time point	Event
Initial consultation	Cosmetic concern; PA diagnosed via physical exam and imaging
Preoperative evaluation	Chest CT, echocardiography, routine labs
Day 0	Minimally invasive surgical correction (Jerusalem Technique)
Post-op day 1–6	Recovery; monitored for complications; pain management, no transfusions
1-month follow-up	Incision healed, high satisfaction reported, resumed normal activity
Ongoing	Planned bar removal after 3 years

## Patient information

The first patient was a 21-year-old male who presented with aesthetic concerns due to a chest wall deformity. He had no significant past medical history, comorbidities, or family history of thoracic deformities.

The second patient was a 13-year-old female, also presenting with cosmetic dissatisfaction. She was otherwise healthy, with no notable psychosocial or genetic concerns. Neither patient had undergone previous interventions related to their deformities.

## Clinical findings

**Figure 1:**
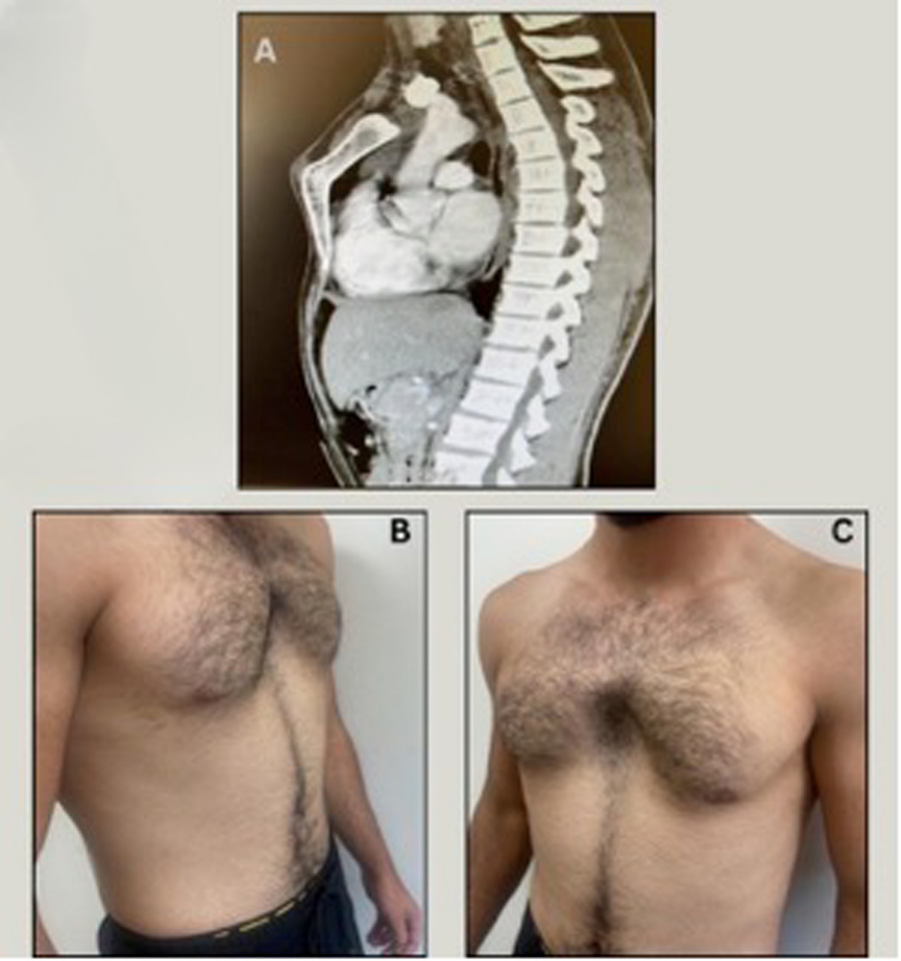
**(A)** Pre-operative CT lateral view | case 1. **(B)** Pre-operative lateral view | case 1. **(C)** Pre-operative anterior view | case 1.

**Figure 2:**
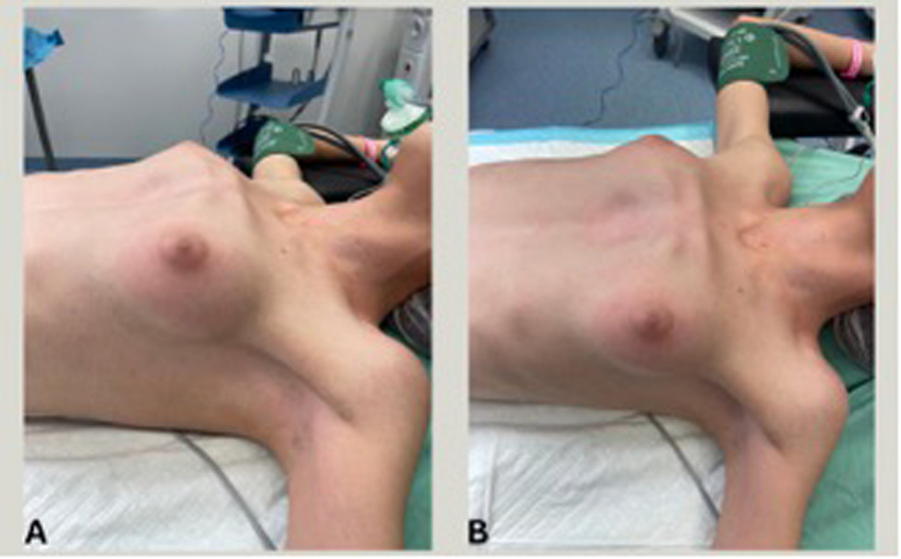
**(A)** Supplementary. Pre-operative lateral view | case 2. **(B)** Supplementary. Pre-operative anterior view | case 2.

Physical examination in both cases revealed the hallmark features of PA: a protruding manubrium and a concave mid-to-lower sternum (Figures 1, 2). No cardiopulmonary symptoms were reported. Both patients had normal physical and developmental findings aside from the thoracic malformation.

## Diagnostic assessment

Preoperative chest CT imaging demonstrated the characteristic S-shaped configuration of the sternum. Echocardiography and laboratory evaluations were unremarkable. The primary diagnostic challenge was differentiating PA from more common chest wall deformities. Following thorough evaluation, the diagnosis of Pectus Arcuatum was confirmed. Prognostically, both patients were expected to recover fully with appropriate surgical correction.

## Therapeutic intervention

Both patients underwent a minimally invasive surgical correction under general anesthesia.

The patients were positioned supine for the corrective procedure. Standard draping and skin preparation were conducted, followed by precise measurement of the pectus bar. Four incisions, each measuring 2.5 cm, were strategically made on the mid-axillary line at the third and fifth intercostal spaces bilaterally. The third and fourth ribs were bilaterally prepared and encircled with metal wires. The bar stabilizer was then securely attached to these wires on both sides. Anterior skin tunnels were created from the upper incisions to the angle of Louis, and posterior skin pockets were extended to accommodate the distal end of the pectus bars, ensuring it conformed closely to the chest wall posterior to the midaxillary line.

A 5 mm thoracoscope was inserted through a trocar placed at the right 6th intercostal space along the posterior axillary line, with CO2 insufflation maintained at a pressure of 10 mmHg. Concurrently, two longitudinal parasternal incisions, each 1.5 cm in length, were made at the level of the manubrium. Thoracoscopic guided dissection was performed around the manubrium through these incisions to facilitate osteotomy. A Gigli saw was then maneuvered around the manubrium, executing a transverse osteotomy (Supplementary Video S1).

Subsequently, the guide was positioned subcutaneously under the pectoralis muscles, followed by the placement of the measured “anterior” bar, which was secured to the stabilizer using metal wires and screws. After careful mediastinal dissection in order to achieve the critical view of safety, additional sternal wires were sutured bilaterally to the edges of the manubrium and anchored to the anterior bar.

The pectus introducer was inserted through the lower intercostal space at the apex of the pectus ridge, aligning with the deepest point of sternal depression. Under videoscopic surveillance, the introducer was carefully advanced across the anterior mediastinal space just beneath the sternum, as the instrument traversed to the contralateral side. The tip emerged through the corresponding intercostal space on the left, and an umbilical tape was drawn through this tunnel. The curved side of the pectus bar was attached to the tape, and with thoracoscopic guidance and traction on the tape, the bar was advanced into position. Initially inserted with its convexity posteriorly between the sternum and mediastinum, the bar was then flipped and secured with a single stabilizer on the right side. A second same sized bar was added (but not in the female patient considering deformity severity and specific anatomy) in the same manner utilizing the same incisions.

The body of the sternum was then elevated into a point that meets the new position of the manubrium. The bar ends were positioned subcutaneously, anterior to the muscle fascia.

Closure of all incisions was performed in layers. The patients were extubated on the operating table and transferred to the recovery room in a stable condition. The procedure lasted approximately 3–3.5 h with minimal blood loss (50–60 cc) and no need for transfusion. No intraoperative or postoperative complications occurred.

## Follow-up and outcomes

**Figure 3:**
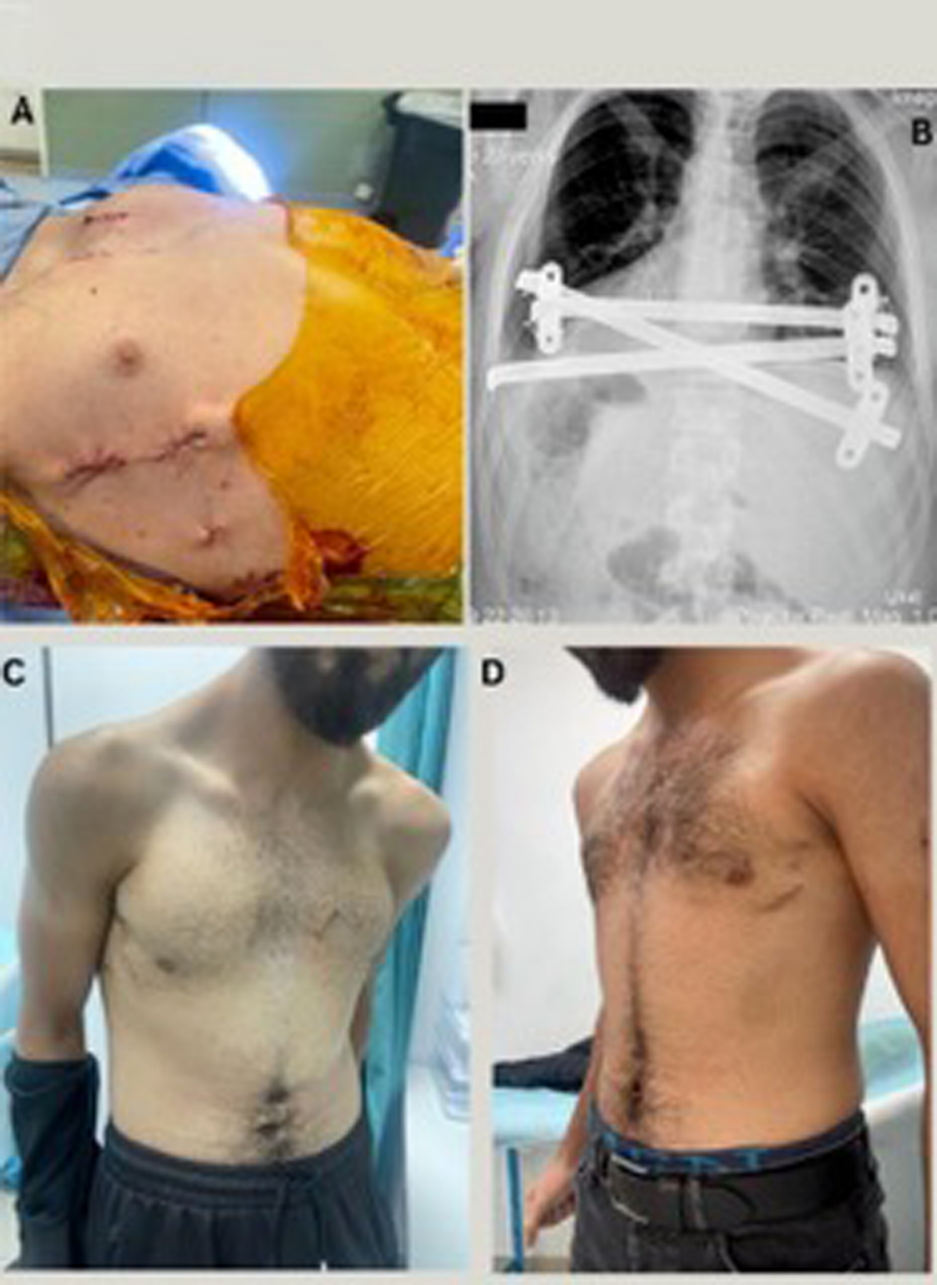
**(A)** Supplementary. Post operative | case 1. **(B)** Supplementary. Post operative x-ray | case 1. **(C)** Supplementary. 30 days postoperative lateral view | case 1. **(D)** Supplementary. 30 days postoperative anterior view | case 1.

**Figure 4:**
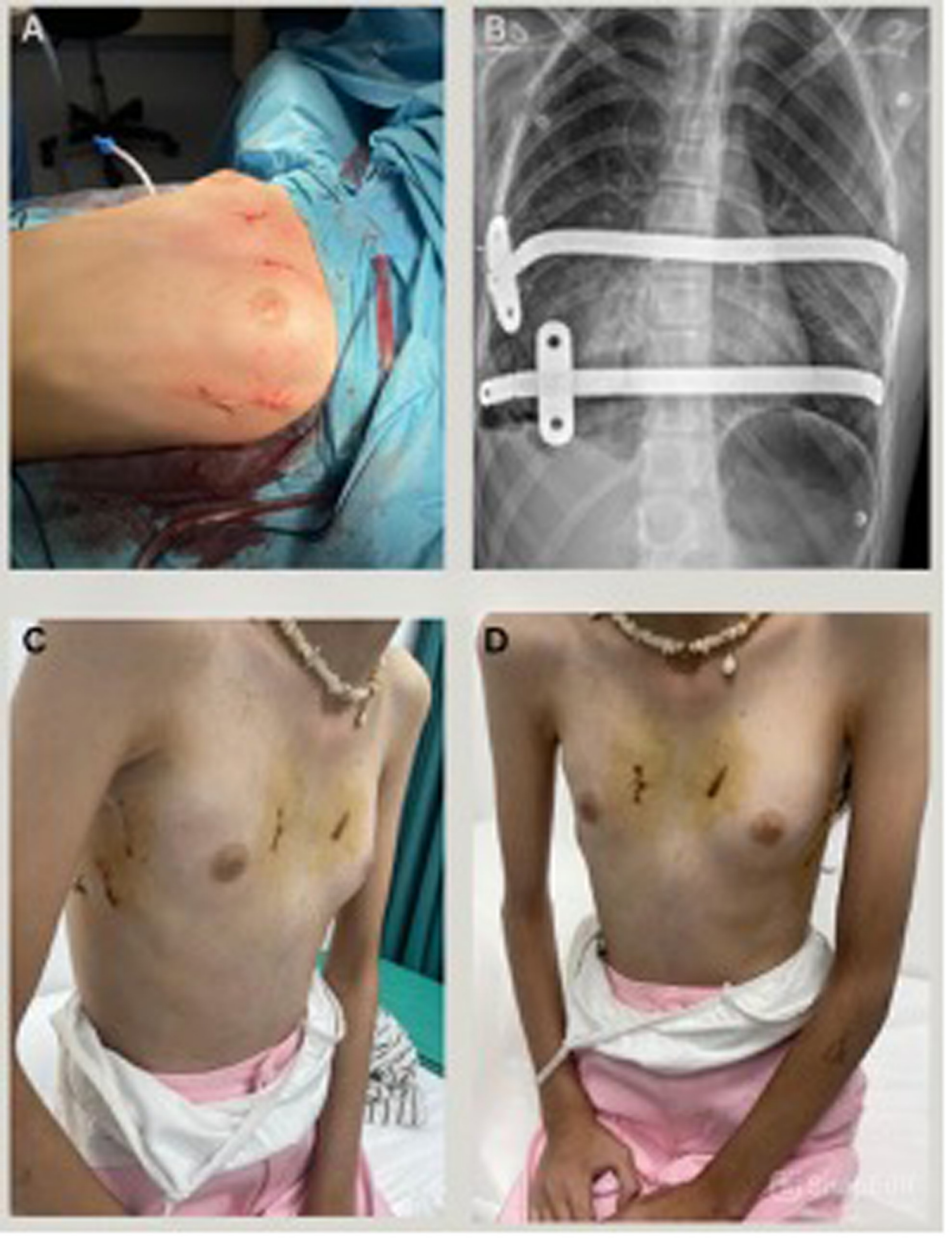
**(A)** Supplementary. Post operative | case 2. **(B)** Supplementary. Post operative x-ray | case 2. **(C)** Supplementary. 30 days postoperative lateral view | case 2. **(D)** Supplementary. 30 days postoperative anterior view | case 2.

Postoperatively, the patients were closely monitored in the recovery room, both patients had minimal pain (average score of 1), rapid recovery, and short hospital stays (4 and 6 days). Early follow-up imaging confirmed optimal positioning of the bars and full correction of the deformity. Both patients returned to normal activities without restrictions. Satisfaction was high, particularly regarding the cosmetic results (Figures 3, 4). There were no adverse events or signs of bar displacement. Planned bar removal is scheduled for three years postoperatively.

## Discussion

Pectus Arcuatum is a rare, poorly classified chest wall malformation often confused with other deformities due to overlapping physical characteristics, initially described by Guido Currarino and Frederic Silverman in 1958 and is commonly referred to as Currarino Silverman (CS) syndrome and Pouter Pigeon Breast. Unlike PE and PC which are the two most common anterior CWM (1), PA additionally involves premature obliteration of the sternum sutures, which lead to angulated synostosis and bilateral deformity of second to fifth costal cartilage.

It has a unique appearance that is characterized by a short, wide, s-shaped sternum, manifested by an outward extrusion of the sternomanubrial junction and a posterior depression of the lower part of the sternum body (1).

Classifying PA has posed significant challenges due to its rarity, leading to a lack of consensus in the literature over the years.

In 2006 Acastello extended the CWM classification by the origin of the malformation. By this classification, “Acastello” subdivided PC into two subtypes: inferior (Chondrogladiolar), and superior (Chondromanubrial) - which is referred to as PA (2). Subsequently, Torre et al. (3) reclassified PA as a sternal anomaly type since it involves the sternum.

In reviewing the literature there isn't a consensus about how PA is classified. Over the years it was described as a subtype of PE, PC, mixed and unusual CWM (1–4, 8).

While generally asymptomatic, PA may present a range of psychological and physiological effects, including low self-esteem and potential pulmonary or cardiac dysfunction (1) which indicate surgical correction.

In 1952, Ravitch described PA as an “unusual sternal deformity with cardiac symptoms”, and published his surgical approach for PA. This method includes chest midline incision, rib cartilage resection and correction of the sternum position when osteotomy performed (4). The Ravitch method and its variations were once the leading approach for correcting chest wall deformities. However, since the late 1990s, minimally invasive surgical techniques, like the Nuss procedure (11) for PE and the Abramson technique (12) for PC, have become the predominant methods.

While minimally invasive procedures have emerged as the predominant technique for repairing CWM, they are not commonly employed for PA due to the necessity of sternum osteotomy. Consequently, the predominant methods for PA correction continue to be the Ravitch technique and its modifications (5–7). Nevertheless, an innovative “hybrid” approach, combining the modified Ravitch technique and the Nuss procedure and hybrid techniques for the correction of PA, was developed and published between 2014 and 2024. Reports indicate the successful implementation of this approach in 14 patients (8–10, 13, 14).

To our knowledge, the Modified Sandwich Technique represents the first fully minimally invasive approach to repairing PA. This innovative method combines elements from the Nuss and Abramson procedures, with the specific goal of achieving superior cosmetic results by avoiding midline incision and possibly reducing postoperative pain, recovery time, and improved aesthetic results. The postoperative period was uneventful followed by normal chest x-ray findings. Patients were discharged, on average, after an average of 5 days (4 and 6 days). During the follow-up appointment two weeks post-discharge, the wound was found to be closed and healed (Figures 3, 4) and the patients expressed satisfaction with the aesthetic results.

Pectus Arcuatum is a rare, poorly classified chest wall malformation often confused with other deformities due to overlapping physical characteristics (2, 3). Existing surgical approaches, especially the Ravitch technique, although effective, are invasive and result in visible midline scarring. The minimally invasive Nuss and Abramson techniques have revolutionized treatment for PE and PC, respectively, but have not been widely adopted for PA due to the requirement for osteotomy.

Recently, hybrid procedures have been developed that combine sternotomy with bar support, but these still involve major incisions. The Jerusalem Technique reported here is, to our knowledge, the first fully minimally invasive surgical approach for PA. It successfully avoids midline incisions, reduces recovery time, and offers excellent cosmetic and functional outcomes. The procedure is suitable for both adolescent and adult patients, as demonstrated by the two cases presented.

Strengths of this approach include minimal invasiveness, short recovery time, and high patient satisfaction. Limitations include the small patient sample and the absence of long-term outcome data, which will be addressed in future studies. This novel method offers a promising new standard for treating PA.

## Conclusion

PA deformity is a unique congenital condition within the spectrum of CWM, characterized by its complexity, which necessitates not only cosmetic repair but also sternotomy. The modified Sandwich technique (Jerusalem Technique) offers an innovative solution aimed at improving cosmetic outcomes by relocating the incisions and minimizing surgical trauma when compared to other methods such as the Ravitch and Hybrid procedures. This technique is designed to achieve optimal correction with less visible scarring, thus enhancing the overall aesthetic result.

Our preliminary experience, based on two cases, demonstrated successful outcomes with no significant complications, suggesting the feasibility of this approach. However, while these initial results are promising, further research with a larger cohort of patients is essential to fully assess the technique's safety, efficacy, and long-term outcomes. Such studies would help strengthen the evidence supporting this method and potentially establish it as a viable option for PA correction in clinical practice.

## Patient perspective

Both patients reported high levels of satisfaction with the surgical outcomes. The 21-year-old male noted a significant improvement in self-image and confidence, with minimal scarring. The 13-year-old female and her family expressed strong satisfaction with both the aesthetic result and the return to regular activities.

## Data Availability

The original contributions presented in the study are included in the article/Supplementary Material, further inquiries can be directed to the corresponding author.

## Supplementary material

Supplementary Video S1 A supplementary video demonstrating the sternal osteotomy stage of the surgery.
